# Selective Extraction and Antioxidant Properties of Thiol-Containing Peptides in Soy Glycinine Hydrolysates

**DOI:** 10.3390/molecules23081909

**Published:** 2018-07-31

**Authors:** Xiuzhen Ding, Xiangzhen Kong, Yeming Chen, Caimeng Zhang, Yufei Hua, Xiangyang Li

**Affiliations:** 1 Key Laboratory of Food Processing Technology and Quality Control in Shandong Province, Grain Process Engineering and Technology Center In Shandong Province, School of Food Science and Engineering, Shandong Agricultural University, 61 Daizong Avenue, Taian 271018, China; xzd@sdau.edu.cn (X.D.); xiangyang_l@163.com (X.L.); 2 State Key Laboratory of Food Science and Technology, School of Food Science and Technology, Jiangnan University, 1800 Lihu Avenue, Wuxi 214122, China; chenyeming19821213@163.com (Y.C.); cmzhang@jiangnan.edu.cn (C.Z.); yufeihuajiangnan@126.com (Y.H.)

**Keywords:** thiol-containing peptides, DTT reduction, Thiopropyl-Sephrose 6B covalent chromatography, antioxidant activities

## Abstract

A highly selective procedure to extract thiol-containing peptides (TCPs) from complicated soy glycinin hydrolysates (SGHs) was described. This procedure included the reduction of disulfide bonds by 1,4-dithiothreitol (DTT) and enrichment of TCPs through Thiopropyl-Sephrose 6B covalent chromatography. TCPs were confirmed using a strategy based on mass shift after differential alkylation of sulfhydryl groups with iodoacetamide and *N*-ethylmaleimide by matrix-assisted laser desorption ionization mass spectrometry (MALDI-TOF-MS). The antioxidant activities of TCPs were evaluated using chemical assays. DTT reduction increased the concentration of sulfhydryl groups from 1.8 μmol/g to 113.8 μmol/g. The efficiency of the extraction was improved by optimizing the loading of sample, extraction and desorption time and the content of desorption reagent. Both of the adsorption and desorption process were found to fit a pseudo-second order model. MALDI-TOF-MS showed that 36 of the 45 extracted peptides were TCPs. The EC_50_ of TCPs for DPPH, hydroxyl radical, and superoxide anion radical was 0.1, 1.49 and 0.084 mg/mL, respectively. The reducing power of TCPs (0.2 mg/mL) was of 0.375. These results suggest that the combination of DTT reduction and Thiopropyl-Sepharose 6B covalent chromatograph was a successful pathway to extract TCPs from SGHs and the TCPs could be used as potential antioxidants.

## 1. Introduction

Thiol-containing compounds exert unique properties that could improve food safety, nutrition, and health. Researchers showed that thiols could suppress the mutagenic activity of aflatoxin B_1_ [1] and other mutagens [2]. Thiol groups exhibited great activity to react with the dehydroalanine and thus inhibit the formation of lysinoalanines during the alkali treatment of food proteins [3]. Lysinoalanine has been found to cause histological changes in the descending portion of the proximal tubules of rat kidneys. Thiol-containing compounds were able to improve the bioavailability of legume proteins by the way of formation of mixed disulfide bonds [4]. Some of thiol-containing compounds are used therapeutically to treat diseases, including acrylonitrile intoxication [5], alcohol- and acetaminophen-induced liver and stomach injuries [6,7], liver necrosis induced by the tricyclic antidepressant amineptine [8], oral tumors [9]. In addition, thiol-containing peptides, such as glutathione, phytochelatins and metallothionein, are important detoxification of toxic metals in the organism [10]. Synthesized thiol-containing peptides exhibited potent antioxidant activities [11,12,13]. The great reactivity of thiol-containing compounds results from the chemical properties of sulfhydryl groups [14], including: (a) polarization of outer shell sulfur electrons; (b) the availability of d-orbitals in the electronic structure of sulfur, permitting d-orbital overlap during the formation of transition states; and (c) the ability of sulfur to act as a free-radical trap, whereby free electrons in highly reactive oxygen radicals are transferred or dissipated to sulfur atoms. Consequently, thiol-containing peptides (TCPs) may also possess these diverse bioactivities.

Soy proteins are well known proteins with high nutritional value, functional properties and low cost and thus are widely used in a range of food produces [15]. Moreover, soybean-derived peptides possess many beneficial bioactive properties [16], which increased its popularity in health food products. However, most of the soy proteins are still applied in the feed industry [17], which is in the manner of low-value. Applying soy proteins to food industry (high-value produces) needs further exploration. Soy glycinine has relative contents of cysteine and enzyme hydrolysis may release bioactive thiol-containing peptides. Nevertheless, limited research has aimed at TCPs of 11S hydrolysates until now.

It is necessary to isolate peptides in order to investigating their structure, bioactivity and application. However, isolation of peptides from protein hydrolysates is often challenging. These hydrolysates are complicated mixtures of peptide fragments. Soy glycinin trypsin hydrolysates contain about 210 peptides. Obviously, non-specific proteases will release far more peptides. The traditional strategies involve assay-guided consecutive separation, including membrane filtration, isoelectric focusing, gel filtration, ion exchange and reverse phase chromatography, which need the analysis of activities fraction by fraction [18,19,20,21]_ENREF_14. Although these procedures are feasible, they are time-consuming and labor-intensive and give rise to problems such as poor isolation yields and loss of biological activity during operation. The properties, be readily oxidized by oxygen molecular [22] and low quantities of TCPs in protein hydrolysates, will exert more difficulties to this task. In consequence, more simple and efficient extraction strategy needs to be explored. Thiopropyl-Sepharose 6B covalent chromatography based on the exchange of thiols and disulfides provides a good alternative for the extraction of TCPs. As only sulfhydryl groups undergo thiol-disulfide exchange chemistry, the specificity of this technique is high. Meanwhile, this technique is simple and high-throughput and thus high efficiency. Furthermore, the reversible capture and release reaction of this method for TCPs generates no side reactions. It has been successfully applied in mouse brain proteome [23], mammary epithelial cell of human [24] and myocardial redox of rat [25]. To our best knowledge, Thiopropyl-Sepharose 6B covalent chromatography has limited applications in extraction of TCPs from food protein hydrolysates, the scale of which is larger than that of proteome. Thiopropyl-Sepharose 6B covalent chromatography was used to isolate TCPs from soy glycinin hydrolysate in our previous work [26], but the isolation method was not investigated in detail. Therefore, the application of Thiopropyl-Sepharose 6B covalent chromatography in extraction of TCPs from food protein hydrolysates to discover novel bioactive peptides needs further research.

The main objective of this research was giving a rapid and efficient procedure to selectively extract TCPs from complicated soy glycinin hydrolysates. Reduction of disulfide bonds was reduced to sulfhydryl groups to improve the recovery of the peptides. The extraction conditions, including sample loading, binding time, release time and the concentration of release reagent, were carefully optimized. The sulfhydryl groups of the obtained TCPs were confirmed by MALDI-TOF-MS based on the difference between alkylation of thiol groups with iodoacetamide and N-ethylmaleimide. The antioxidant activities of TCPs were also analyzed.

## 2. Results and Discussion

### 2.1. Reduction of Disulfide Bonds in SGHs

**Figure 1:**
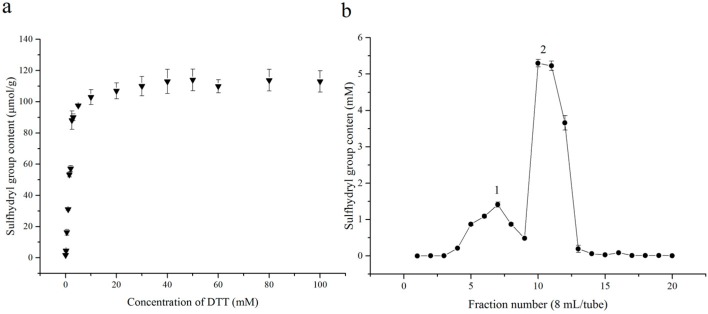
(**a**) Effect of concentration of DTT on the sulfhydryl group content of SGHs; (**b**) Sephadex LH-20 profile of reduced SGHs.

The content of sulfhydryl groups in purified glycinin was 1.4 mol/mol and increased to 41 mol/mol after full reduction [27]. Namely 96.6% cysteine residues were in the form of disulfide bonds. The content of sulfhydryl groups decreased a little during the hydrolysis due to oxidation according to our previous studies (unpublished data). Consequently, it is necessary to reduce disulfide bonds to sulfhydryl groups in order to obtain more TCPs. DTT was the most commonly used reductant and could release two sulfhydryl groups from one disulfide bond after which it was converted to a stable intra-molecular cyclic disulfide [28]. The main advantage of this reductant is the high degree of specificity to disulfides but main disadvantage is that the SH groups could compete directly with the sulfhydryl groups in SGHs in the latter procedure. Figure 1a shows the effect of DTT on the reduction of disulfide bonds. The sulhydryl group values increased rapidly with the increase of DTT concentration when the concentration was between 0–5 mM and leveled off at a higher DTT concentration. The content of sulfhydryl showed no significant (*p* < 0.05) different when the concentration of DTT increased from 30 to 100 mM. It was indicated that 30 mM DTT was sufficient to reduce the disulfide bonds in SGHs. However, it was noticed that the content of DTT was excess in comparison to the sulfhydryl groups in the fully reduced SGHs. Therefore, some treatment was needed in order to remove the excess DTT.

**Figure 2:**
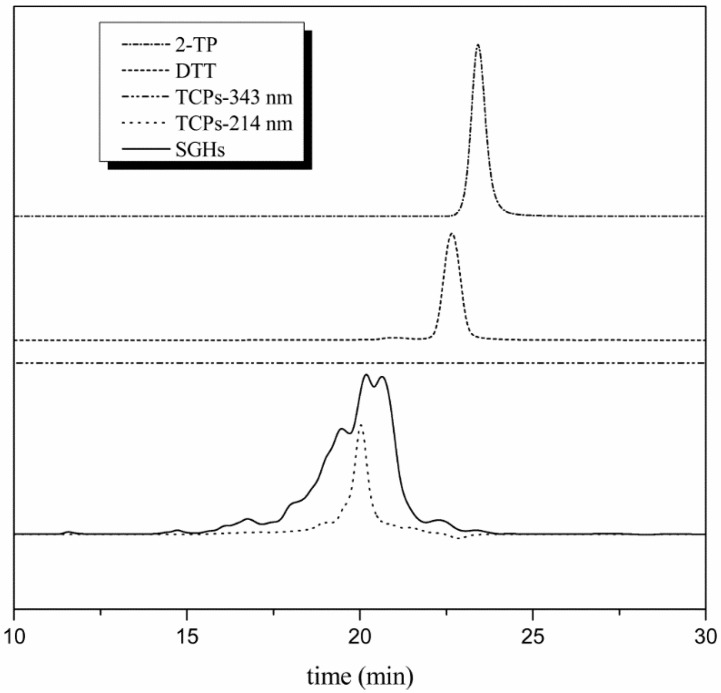
SEC-HPLC profile of reduced SGHs and TCPs. The Y axis was the absorbance of the elution at various wavelength and was omitted for the convenience of comparison between different compounds.

A Sephadex LH-20 column was applied in order to remove the excess DTT [29]. 1 mM HCl was used to elute the sample where no oxidation of sulfhydryl groups was expected. The profile of Sephadex LH-20 was as shown in Figure 1b. Reduced SGHs (Pick 1) was separated well with DTT (Pick 2). At pH 3.0 peptides were positively charged, while DTT was neutral. So SGHs were eluted in the void column regardless of size, whereas DTT was retarded. SEC-HPLC (Figure 2) showed that no DTT was detected in reduced SGHs. The content of sulfhydryl groups was analyzed using 4,4′-dithiodipyridine (4-DPS) before and after reduction. The content of sulfhydryl groups was 1.8 μmol/g and increased to 113.8 μmol/g after reduction, with 63.2 folds increase being obtained. These values agreed well with the results of Wolf [27] according the molecular weight of 11 S being 320,000 Da and purity of 11 S being 91%. The results suggested that DTT reduction and Sephadex LH-20 column separation was an effective pathway to increase the content of sulfhydryl groups in SGHs.

### 2.2. Optimization of the Extraction Conditions of TCPs

#### 2.2.1. Optimization of the Capture Conditions of TCPs

**Figure 3:**
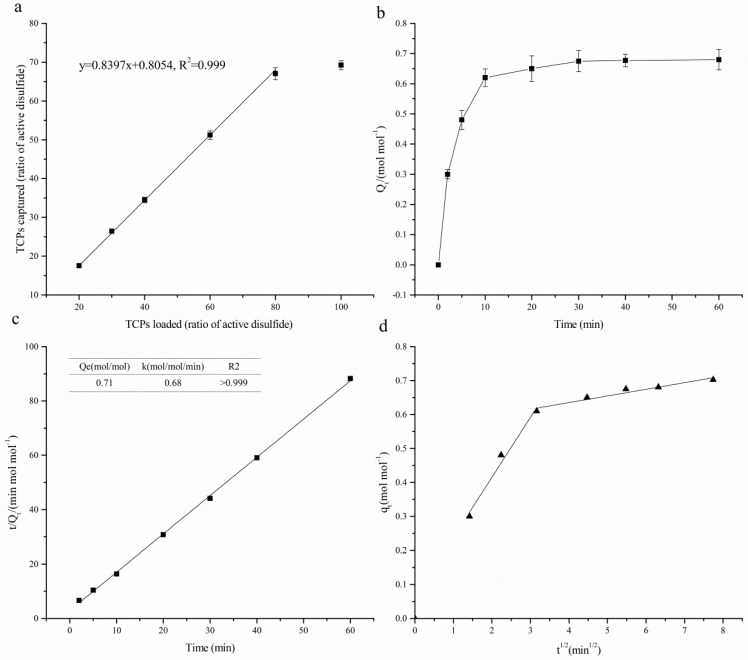
(**a**) effect of dosage of SGHs on the capture of TCPs by Thiopropyl Sepharose 6B; (**b**) contact time curve of the capture of TCPs; (**c**) plots of the pseudo-second order linearized kinetic model for the capture of TCPs; (**d**) plots of the Weber-Morris intra-particle diffusion for the capture of TCPs.

TCPs in reduced SGHs were captured by covalent chromatography using Thiopropyl-Sepharose 6B. In order to find the optimum amount of the resin for maximizing the interactions between TCPs and capture sites of the resin, various amounts of TCPs (expressed as the concentration of sulfhydryl groups: 20%, 30%, 40%, 60%, 80% and 100% of the active disulfide) were added to 0.5 g resin (35.5 μmol active disulfide per gram rein) and the mixture was shaken for 2 h. As shown in Figure 3a, TCPs captured by the resin was increased with the dosage loading within 20–80% of active disulfides of the resin. 100% loading did not show significant difference with that of 80%, which indicated that loading of 80% of active disulfides was suit for the extraction. A strongly positive linear correlation (r > 0.999) was observed with a low variance (R^2^ > 0.999) and a slope close to unity (0.84), indicating an almost equimolar reaction between peptides and resin at loadings of less than 80% of the active disulfides. Paulech et al. [25] discovered similar positive linear correlation, however, the slope was 1:1.186 mol Cys: 2-TP, which was induced by the high reactivity of peptides containing vicinal thiols to the active disulfide bonds.

Figure 3b shows the capture kinetic curve of TCPs, expressed as concentration of sulfhydryl groups, by Thiopropyl-Sepharose 6B. As can be seen, the curve appeared rapid rising at the initial 10 min and then approaching flattening at 30 min. So that, the optimal equilibrium time was 30 min. This length of time was much less than the capture of thiol-containing proteins. The rapid capture in the first minutes can be attributed to the availability of a large number of vacant surface sites of the gel. The decreasing capture rate is perhaps due to the slow pore diffusion of TCPs into the bulk of the gel. The data were found to fit pseudo-second order model Equation (1) with high regression coefficient (R^2^ > 0.999) (Figure 3c). The concentration of captured sulfhydryl groups at equilibrium (Qe) was 0.71 mol/mol active disulfides (Figure 3c):

(1) $$\frac{t}{Q_{t}}=\frac{1}{k\cdot Q_{e}^{2}}+\frac{1}{Q_{e}}t$$

where, Q_t_ and Q_e_ are the concentrations of captured sulfhydryl groups (mol·mol^−1^) at contact time t (min) and equilibrium, respectively, and k is the pseudo-second order kinetic parameter (mol·mol^−1^·min^−1^).

The possibility of intra-particle diffusion resistance affecting adsorption was explored by using the intra-particle diffusion model as:

(2) $$q_{t}=k_{\mathrm{id}}t^{1/2}+I$$

where, k_id_ is the diffusion rate constant. Values of *I* give an idea about the thickness of the boundary layer, i.e., the larger intercept the greater is the boundary layer effect.

In Figure 3d, a plot of TCPs adsorbed per unit molar of active sit, q_t_ versus t^1/2^ is presented. The plots show that the adsorption processes consist of two linear sections with different slopes, indicating that two diffusion steps occurred in the adsorption process. The first portion of the straight line represents the diffusion process controlled by external surfaces, and the second portion of the straight line shows the intra-particle diffusion. The intra-particle diffusion was the rate-limiting step. However, the capture process was not controlled solely by the intra-particle diffusion since the extrapolation of the second linear portion did not pass through the origin [30]. External mass transfer or film diffusion may be involved in the capture process.

#### 2.2.2. Optimization of the Desorption Conditions of TCPs

TCPs captured by the Thiolpropyl-Sepharose 6B were released by DTT in 10 mM pH 8.0 Tris-HCl buffer at 25 °C. The concentration of DTT in the buffer and incubating time was optimized with regard to desorption yield of TCPs in order to improve the efficiency. Desorption yield of TCPs was expressed as the percentage of captured TCPs which was presented as the area of the SEC-HPLC profile.

**Figure 4:**
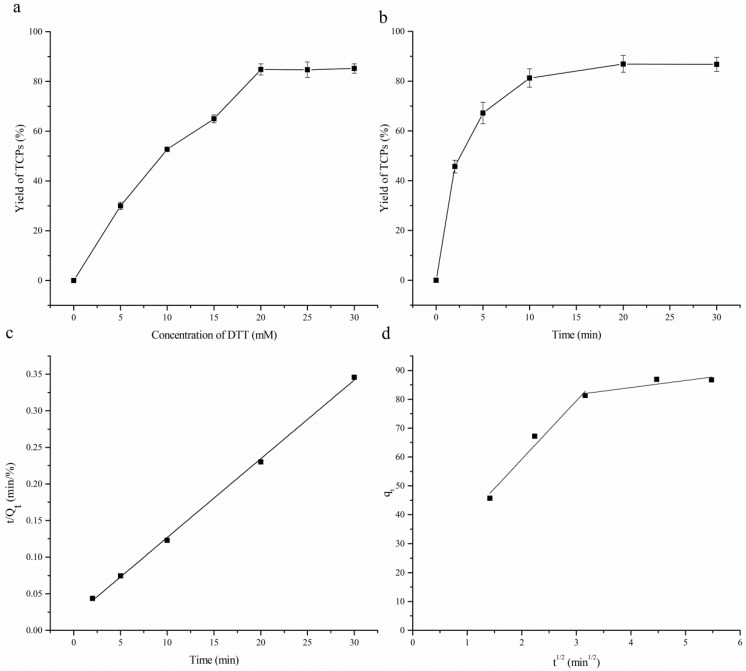
(**a**) Effect of concentration of DTT on the desorption yield of TCPs; (**b**) effect of incubating time on the desorption yield of TCPs; (**c**) plots of pseudo-second order kinetic model for the desorption of TCPs; (**d**) plots of the Weber-Morris intra-particle diffusion for the desorption of TCPs.

The effect of concentration of DTT was presented in Figure 4a. The yield of the TCPs was increased significantly (*p* < 0.05) in a linear manner as the concentration of DTT increased from 5 mM to 20 mM. 84.8% of TCPs was recovered by 20 mM DTT. Meanwhile no significant change of the yield was observed with further increase of the dosage of DTT. These results indicated that 20 mM DTT in 10 mM pH 8.0 Tris-HCl buffer was sufficient to release the TCPs captured by the resin. The loss of other TCPs may be due to the retention of pores among the resin. According to the SEC-HPLC profiles of SGHs before enrichment and TCPs obtained (Figure 2), 3.2% of the hydrolysates were recovered.

The effect of incubating time on the recovery yield of TCPs is shown in Figure 4b. Similar to the capture of TCPs, the yield of TCPs increased significantly (*p* < 0.05) in the initial 10 min. This increase was attributed to the high affinity of DTT to disulfide bonds between the active site and the TCPs on the surface of the resin. At 10 min to 30 min, the yield of TCPs increased further but with a small growth rate. TCPs in this stage may come from the interior of the pores among the resin. The kinetic of the release of TCPs was fit well to the pseudo-second order model (Figure 4c) with high regression coefficient (R^2^ > 0.999). The plots of intra-particle diffusion model (Figure 4d) indicated that desorption of TCPs contained two steps, namely external surfaces diffusion and intra-particle diffusion. The intra-particle diffusion was the rate-limiting step. The extrapolation of the second linear portion does not pass through the origin demonstrated that desorption of TCPs was not controlled solely by the intra-particle diffusion.

In previous studies, various concentrations of DTT and incubation times had been employed to release TCPs captured by Tiolpropyl-Sepharose 6B. Liu et al. [24] applied 20 mM DTT to incubate 30 min. 25 mM DTT was used by Wang et al. [23]. And 5 × 10 mM DTT [25] combined with 15 min incubating was also employed. In our optimization, 20 mM DTT combining with incubating time of 10 min was the most effective and economic.

#### 2.2.3. Purification of TCPs by Reversed Phase Chromatography Column

TCPs released from Thiopropyl-Sephrose 6B contained DTT and 2-thiopyridone (2-TP). A C18 column was applied to remove these contaminates. It was noticed that DTT and 2-TP were separated from TCPs simultaneously. As shown in Figure 2, DTT had relative absorbance at 214 nm with eluting time being 22.5 min at SEC-HPLC profile. 2-TP had intense absorbance at 343 nm at 23.7 min at SEC-HPLC profile. The SEC-HPLC profiles of TCPs at 214 nm and 343 nm, respectively, were also given. As can be seen, the picks of TCPs at 214 nm were almost focusing on 18–22 min. The signal of TCPs at 22.5 min was very small. These results indicated that DTT in TCPs was almost removed by reversed phase chromatography. The profile of TCPs at 343 nm was at baseline, which demonstrated that 2-TP was totally removed. Reverse phase liquid chromatography is the separation of molecules based upon their inter-action with a hydrophobic matrix (C18). When water was the mobile phase, the interaction between TCPs and the matrix was stronger than that between TCPs and water. So TCPs were absorbed on the matrix, while DTT and 2-TP were in the opposite manner. Consequently, DTT and 2-TP were separated from TCPs. And when the mobile phase was changed to 80% acetonitrile, the interaction between TCPs and 80% acetonitrile was stronger than that between TCPs and the matrix. Therefore, TCPs were transferred to the mobile phase. In proteomics, SPE C18 column was often used to desalt before RP-HPLC. The content of sulfhydryl groups of the resultant TCPs was 893.7 μmol/g.

### 2.3. Specificity to TCPs

The specificity of Thiopropyl-Sephrose 6B toward TCPs was determined. Cys-peptides and non-Cys-peptides in TCPs were confirmed using a strategy based on the difference in mass transfer after treatment by iodoacetamide (IAM) and *N*-ethylmaleimide (NEM), respectively, and the mass was determined by MALDI-TOF-MS. The mass of non-Cys-peptides, no reaction with alkylating agents, will not change after the two treatments. However, the mass of one Cys will increase by 57.06 after reaction with IAM, and by 125.13 with NEM. So the distance in mass is 68.07 for one cys-containing peptides and *n* × 68.07 for *n* cys-containing peptides between the two treatments. Using MS techniques, peptide fragments with no thiols are recognized in peptide mass maps according to the same value and peptide fragments containing thiols with modifications are recognized according to an exact mass shift from the expected theoretical values. MALDI-TOF-MS results (Table 1) showed that 9 peptides did not change in mass after the two treatments, there were 32 peptides with mass distance being 68.07 ± 0.5 Da, 4 peptides with mass distance being 2 × 68.07 ± 0.5 Da. Therefore, the extracted TCPs contained 9 non-cys peptides, 32 one-cys-containing peptides and four two-cys-containing peptides. 36 of 45 peptides contained Cys. These results indicated that Thiopropyl-Sephrose 6B exhibited high selectivity to thiol-containing peptides.

### 2.4. Antioxidant Activities of TCPs

The antioxidant activities of TCPs were evaluated by scavenging activity of different radicals, including DPPH, hydroxyl and superoxide anion, and reducing power. The scavenging of DPPH radical of TCPs was found to be a concentration-dependent manner. The EC_50_ value of TCPs for DPPH radical scavenging activity was 0.1 mg/mL (Table 2). This value is significantly (*p* < 0.05) higher than that of glutathione (0.03 mg/mL), but obviously lower than that of enzymatic rapeseed protein [31], chickpea protein hydrolysate [32], germinated legumes [33] and 70% ethanol extracts of mung bean [34]. The scavenging activity of TCPs indicated that the TCPs were able to effectively scavenge the radical of DPPH. The mechanism of this scavenging is that when DPPH encounters a proton-donating substance (H^+^), the radical accepts an electron to become a stable reduced DPPH-H. The scavenging of hydroxyl radical is important for protection the body against various diseases which were caused by the oxidative stress resulting from this radical. Meanwhile, the superoxide radical is a highly toxic radical species that is a precursor of some other highly reactive species such as hydrogen peroxide and hydroxyl radical [35]. Similar to the scavenging of DPPH radical, the scavenging of hydroxyl and superoxide radicals of TCPs were also observed to be a concentration-dependent manner. The EC_50_ value was 1.49 mg/mL and 0.084 mg/mL for hydroxyl and superoxide radicals, respectively. The scavenging abilities were not as effective as glutathione, but comparatively better than chicken skin protein hydrolysates [36] and egg white protein hydrolysates [37], so TCPs exhibited excellent antioxidant activities against hydroxyl and superoxide radicals, which indicated that it could be used as the scavenging agent to reduce hydroxyl and superoxide radicals-induced damage in living body. The free radical chain reactions will be interrupted when unpaired free radicals get electrons to become paired. The reducing power test is often applied to appraise the ability of natural antioxidants to donate an electron or hydrogen. In this experiment, the reducing power of TCPs was investigated at different concentrations (from 0.1 to 0.8 mg/mL), and the reducing power dramatically increased with the increase of TCPs concentration. Many researchers have found that there was a direct correlation between antioxidant activity and reducing power. The reducing ability of egg white protein hydrolysates was 0.215 at the concentration of 5 mg/mL [37], 0.2 and 0.14 at 0.2 mg/mL for walnut [38] and eggshell membrane [39] protein hydrolysates, respectively.

It is reported that the antioxidant activities are closely related to the structure of the peptides among which amino acid composition and size of peptides are considered to be the most important factors. Peptides contained Tyr, Met, His, Lys, Cys and Trp were generally accepted to be strong antioxidants. Huang et al. [13] and Han et al. [11] found that thiol-containing peptides exhibited antioxidant activities. High content of cysteine residues of TCPs in this study may contribute to the antioxidant activities due to their direct reaction with radicals [40]. Most peptides purified from enzymatic hydrolysates are in the size of 2–20 amino acids and peptides less than 6000 Da are most possibly to show antioxidant activity [41]. The structurally unique linkage type of peptide chain has been demonstrated to influence antioxidant capacity [12].

## 3. Materials and Methods

### 3.1. Materials

Low-denatured, defatted soybean meal (protein: 53.13%, nitrogen solubility index: 87%) was given by Shandong Gushen Industrial & Commercial Co., Ltd. (Dezhou, Shandong, China). β-mercaptoethanol was bought from Shanghai Shengzheng Biotech. Co., Ltd. (Shanghai, China). Alcalase 2.4 L FG (180,000 U/mL) was bought from Novozymes (Beijing, China) Biological Technology Co. 4-DPS was bought from Sigma-Aldrich Trading Co., Ltd. (Shanghai, China). DTT and C18 resin were from Merck (Darmstadt, Germany). Sephadex LH-20 and Thiopropyl-Sepharose 6B resins were bought from GE Healthcare Life Sciences (Boston, MA, USA). Chromatographic grade acetonitrile was bought from J&K China Chemical Ltd. (Shanghai, China). Other agents were of analytical grade.

### 3.2. Preparation of Soy Glycinin (11 S)

Soy glycinine (11 S) was prepared from defatted soybean meal in according with Wolf [27] with some modifications. The defatted soybean meal was dispersed in a 30 mM Tris-HCl buffer with a meal to buffer ratio being 1:10 (*w*/*v*) and stirred at room temperature for 1 h. The buffer was at pH 8.0 and contained 10 mM 2-mercaptoethanol. Then the mixture was centrifuged (30 min, 12,000× *g*, 4 °C) to obtain the supernatant, after which the supernatant was adjusted to pH 6.4 with 2 M HCl to precipitate the protein. The precipitated protein was separated by centrifugation (20 min, 12,000× *g*, 4 °C), and then dispersed in distilled water and adjusted to pH 8.0 with 2 M NaOH, and finally dialyzed thoroughly against deionized water to remove the salt and 2-mercaptoethanol. The pH of the dialyzed protein dispersion was adjusted to 8.0 with 2 M NaOH, lyophilized and stored at 4 °C before use. The content of protein of 11 S was 95.3 (±1.6)% (*w*/*w*) was measured by the micro-Kjeldahl method with 6.25 as the nitrogen conversion factor.

### 3.3. Enzymatic Hydrolysis of 11 S

5% (*w*/*w*) 11 S solution was digested at pH 8.0, 50 °C for 6 h by alcalase with an enzyme substrate ratio (*E*/*S*) of 5:100. The hydrolysis was controlled by pH-stat method with 0.5 M NaOH as the titrant. The protease in the final hydrolysates was inactivated by boiling for 5 min. The resultant hydrolysates were centrifuged at 10,000× *g* for 15 min, and then the supernatant was freeze-dried and kept at 4 °C until further analysis.

### 3.4. Reduction of Disulfide Bonds in Soy Glycinine Hydrolysates (SGHs)

The disulfide bonds in soy glycinin hydrolysates (SGHs) (50 g/L) were reduced in 10 mM Tris-HCl buffer at pH 8.0 using different concentrations of 1,4-dithiothreitol (DTT) varying from 0.2 mM to 100 mM. [26] And the reduction was performed at 50 °C for 30 min, after which the pH was reduced to 3.0 by 2 M HCl. Then the solution was centrifuged at 10,000× *g* for 15 min and filtered through 0.22 μm filters (Φ 50 mm × 0.22 μm, low protein binding, Sinopharm Chemical Reagent Co., Ltd. (Shanghai, China)) to remove the insoluble particles. The clear solution was subsequently applied to Sephadex LH-20 column (45 cm × 1.2 cm i.d.) which was extensively washed and eluted using 1.0 mM HCl to remove the excess DTT.

### 3.5. TCPs Enrichment from Reduced SGHs

TCPs in the reduced SGHs were extracted by batch-based Thiopropyl-Sepharose 6B covalent chromatography [25]. Thiopropyl-Sepharose 6B beads were rehydrated and extensively washed using deionized water. Samples eluted from Sephadex LH-20, after adjusted to pH 7.5 and added 0.5% SDS, were immediately applied to the Thiopropyl-Sepharose 6B beads and allowed to react with the beads at room temperature with gentle tumbling in the protection of purified nitrogen. And then the resin which had bound TCPs was cleaned with 2 bed volumes of 0.5% SDS and 10 bed volumes of water to remove the unbound hydrolysates. At last, the bound peptides were liberated using DTT in 10 mM Tris-HCl (pH 8.0) at room temperature. The resulting solution was called crude TCPs.

### 3.6. Reversed Phase Chromatography

TCPs released from Thiopropyl-Sepharose 6B were separated further to remove excess DTT and 2-TP by reversed phase chromatography. The crude TCPs were pooled to a C-18 column (1.0 × 5.0 cm) that had been equilibrated with 0.5% trifluoroacetic acid (TCA). The column was washed with 0.5% TCA until the absorbance of the eluate at 214 nm down to zero. TCPs were eluted by 80% acetonitrile containing 0.5% TCA. Fractions which had absorbance at 214 nm was collected, concentrated by N_2_ flow, lyophilized and stored at −20 °C until use.

### 3.7. Sulfhydryl Group Content Measurement

The content of sulphydryl groups was evaluated using 4-DPS in accordance with Riener et al. [42] with some modification. An aliquot of 0.3 mL sample solutions was firstly mixed with 2.7 mL 0.1 M citrate-Na_2_HPO_4_ buffer (pH 4.5) containing 1% SDS and then 125 μL 4-DPS (4 mM). The mixture was allowed to react at room temperature for 30 min, after which the absorption at 324 nm was recorded. 21,200 M^−1^·cm^−1^ was used as the extinction coefficient. The content of protein was determined by the biuret method with SGHs as the standard. The protein content of SGHs was determined by Kjeldahl Method.

### 3.8. Size-Exclusion High Performance Chromatography (SEC-HPLC)

The TCPs was separated by a TSK gel 2000 SW_XL_ column (300 mm × 7.8 mm i.d., Tosoh, Tokyo, Japan) with an Elite L-2000 HPLC system (Hitachi, Tokyo, Japan). The column was eluted using acetonitrile/water/trifluoroacetic acid = 45/55/0.1 (*v*/*v*) at a flow rate of 0.5 mL/min and the elution was detected at 214 nm. The calibration curve was made according to the average retention times of the molecular weight standard containing cytochrome C (12,500 Da), bacitracin (1450 Da), tetrapeptide GGYR (451 Da) and tripeptide GGG (189 Da).

### 3.9. Alkylation of Sulfhydryl Groups and Matrix-Assisted Laser Desorption/Ionization—Time-of-Flight Mass Spectrometry (MALDI-TOF-MS)

TCPs released from the resin were alkylated using iodoacetamide (IAM) and *N*-ethylmaleimide (NEM), respectively. TCPs with 25 mM DTT in 10 mM Tris-HCl (pH 8.0) was divided into two parts. One was mixed with 80 mM IAM, and the mixture was incubated 20 min in the dark at ambient temperature. Another part was incubated with 80 mM NEM for 15 min at ambient temperature.

In the final step before MALDI-TOF-MS analysis, 0.7 μL of alkylated TCPs was loaded on MALDI target, after air-dried 0.7 μL of 4-hydroxy-α-cyanocinnamic acid or 2,5-dihydroxybenzoic acid was loaded and air-dried. The mass spectra were obtained with an ultrafleXtreme MALDI-TOF-MS (Bruker Daltonics, Karlsruhe, Germany). All mass spectra were analyzed by FlexAnalysis software. Peaks with mass derivations less than 0.5 Da were accepted as matches.

### 3.10. Antioxidant Activities of TCPs

#### 3.10.1. Scavenging Activity against DPPH Radical

The decolourization of TCPs on the DPPH radical solution was estimated according to the method of Huang et al. [13]. An aliquot of TCPs (1 mL) in 100 mM Tris-HCl buffer (pH 7.4) and then 1 mL of DPPH in ethanol with a final concentration of 50 μM was added. The mixture was shaken vigorously and left to stand at room temperature for 20 min in the dark. The absorbance at 517 nm of the reaction solution was measured spectrophotometrically. The percentage of DPPH decolourization of the sample was calculated according to the equation:

(3) $$\mathrm{DPPH}\;\mathrm{radical}\;\mathrm{scavenging}\;\mathrm{activity}\;\left(\%\right)=1-\frac{A_{\mathrm{sample}}{-A}_{\mathrm{blank}}}{A_{\mathrm{control}}}\times 100$$

where A _sample_, A _blank_, and A _control_ are the absorbance of sample, blank and control, respectively.

#### 3.10.2. Scavenging Activity against Hydroxyl Radical

The hydroxyl radical-scavenging activity was measured in accordance with the method of Li et al. [32]. Aliquots of 0.5 mL of 0.75 mM 1,10-phenanthroline, 1 mL of 0.2 M phosphate buffer (pH 7.4), 0.5 mL of sample, and 0.5 mL of 0.75 mM FeSO_4_ were put into a text tube. After mixing, 0.5 mL of 0.01% H_2_O_2_ was added, and the solution was kept at 37 °C for 1 h. Finally, the absorbance of the result solution was detected at 536 nm. The control group was prepared in the same procedure as the sample group except that deionized water was put instead of sample solution. And the blank group was prepared in the same procedure as the control group except that deionized water was added replacing H_2_O_2_ solution:

(4) $$\mathrm{Hydroxyl}\;\mathrm{radical}\;\mathrm{scavenging}\;\mathrm{activity}\;(\%)=\frac{A_{\mathrm{sample}}{-A}_{\mathrm{control}}}{A_{\mathrm{blank}}-A_{\mathrm{control}}}\times 100$$

where A_sample_, A_control_ and A_blank_ are the absorbance of the sample, control, and blank group, respectively.

#### 3.10.3. Superoxide Anion Scavenging Activity Assay

The scavenging activity on superoxide anion was evaluated as the ability to inhibit the auto-oxidation of pyrogallol [37]. An aliquot of 0.1 mL of sample was mixed with 2.8 mL of 0.1 M Tris–HCl buffer (pH 8.2) and the mixture was kept at 25 °C for 10 min, and then 0.1 mL of 3 mM pyrogallol in 10 mM HCl was added. And immediately, the absorbance of the mixture at 320 nm was recorded till 4 min. The oxidation rate of pyrogallol was expressed as the slope of the absorbance line (r_1_). The auto-oxidation rate of pyrogallol of the control (r_0_) was measured using 0.1 mL of deionized water replacing the sample solution. The superoxide anion scavenging activity was calculated as:

(5) $$\mathrm{Superoxide}\;\mathrm{anion}\;\mathrm{scavenging}\;\mathrm{activity}\;\left(\%\right)=\frac{r_{0}-r_{1}}{r_{0}}\times 100$$

#### 3.10.4. Reducing Power Measurement

The ability of TCPs to reduce iron (Fe^3+^) was measured according to the method of Chen et al. [37] with some modifications. Aliquots of 2 mL of sample, 2 mL of 0.2 M phosphate buffer (pH 6.6) and 2 mL of 1% (*w*/*v*) potassium ferricyanide were pipetted into a text tube and kept at 50 °C for 20 min, after which 2 mL of 10% (*w*/*v*) trichloroacetic acid (TCA) was added. And the mixture was then centrifuged at 3500× *g* for 10 min. In the end, 2 mL of the supernatant was mixed with 2 mL of deionized water and 0.4 mL of 0.1% (*w*/*v*) ferric chloride. After 10 min of standing at 50 °C, the absorbance of the resulting solution was measured at 700 nm.

### 3.11. Statistical Analysis

Data were shown as the mean ± SD (*n* = 3) analyzed by analysis of variance (ANOVA) using the SPSS 20.0 software (IBM, Armonk, Ney York, NY, USA). A least significant difference (LSD) test with a confidence interval of 95% was used to compare the means. All treatments were run in triplicate.

## 4. Conclusions

In conclusion, the procedure that combined DTT reduction and Thiolpropyl-Sepharose 6B covalent chromatograph successfully applied in the extraction of TCPs in SGHs. DTT reduction increased the content of sulfhydryl groups by 63.2 times. Thiolpropryl-Sepharose 6B covalent chromatograph showed high selectivity to TCPs, which was much faster than the traditional multiple-step chromatography. In addition, TCPs extracted presented potent antioxidant activities using chemical assays. It is of great importance for the improvement of soy protein value and increasing the income of the peasants.

## Figures and Tables

**Table 1:** *m*/*z* of picks in MALDI-TOF-MS spectrum.

Pick Number	IAM	NEM	Distance in *m*/*z*	Content of Cys
1	198.774	198.769	−0.005	0
2	274.156	274.149	−0.007	0
3	284.22	284.222	0.002	0
4	312.92	312.911	−0.009	0
5	318.22	318.202	−0.018	0
6	833.497	833.4	−0.097	0
7	905.439	905.408	−0.032	0
8	992.494	992.485	−0.009	0
9	1169.603	1169.569	−0.033	0
10	249.849	318.202	68.353	1
11	315.021	383.135	68.113	1
12	335.026	403.133	68.106	1
13	482.176	550.227	68.051	1
14	517.18	585.244	68.064	1
15	559.205	627.266	68.061	1
16	713.328	781.374	68.046	1
17	765.341	833.408	68.068	1
18	777.363	845.422	68.059	1
19	799.351	867.417	68.066	1
20	815.341	883.404	68.063	1
21	821.341	889.402	68.061	1
22	864.424	932.471	68.047	1
23	910.428	978.458	68.031	1
24	921.453	989.523	68.069	1
25	928.422	996.486	68.064	1
26	940.452	1008.478	68.026	1
27	992.494	1060.551	68.056	1
28	1041.518	1109.589	68.071	1
29	1049.587	1117.515	67.928	1
30	1063.511	1131.587	68.076	1
31	1079.487	1147.567	68.081	1
32	1085.519	1153.529	68.01	1
33	1150.572	1218.581	68.009	1
34	1174.575	1242.592	68.017	1
35	1177.58	1245.652	68.072	1
36	1188.58	1256.645	68.065	1
37	1197.631	1265.685	68.055	1
38	1204.609	1272.617	68.008	1
39	1210.61	1278.59	67.981	1
40	1226.607	1294.613	68.006	1
41	1249.618	1317.626	68.008	1
42	713.328	849.39	136.063	2
43	921.453	1057.523	136.07	2
44	1049.587	1185.595	136.008	2
45	1125.593	1261.639	136.046	2

**Table 2:** Antioxidant activities of TCPs.

	TCPs	Glutathione
DPPH (mg/mL) ^A^	0.10 ± 0.004 ^a^	0.03 ± 0.001 ^b^
Hydroxyl radical (mg/mL)	1.49 ± 0.07 ^a^	0.36 ± 0.014 ^b^
Superoxide anion radical (mg/mL)	0.084 ± 0.004 ^a^	0.052 ± 0.003 ^b^
Reducing power ^B^	0.38 ± 0.01 ^a^	0.57 ± 0.031 ^b^

Different superscript characters (^a^ and ^b^) indicate significant difference at *p* < 0.05 level within the same row. ^A^ EC_50_^B^ Sample concentration was 0.2 mg/mL for reducing power (OD 700 nm).
